# Chronic Exposure to Alcohol Inhibits New Myelin Generation in Adult Mouse Brain

**DOI:** 10.3389/fncel.2021.732602

**Published:** 2021-08-27

**Authors:** Feng Guo, Yi-Fan Zhang, Kun Liu, Xu Huang, Rui-Xue Li, Shu-Yue Wang, Fei Wang, Lan Xiao, Feng Mei, Tao Li

**Affiliations:** ^1^Brain and Intelligence Research Key Laboratory of Chongqing Education Commission, Department of Histology and Embryology, Third Military Medical University (Army Medical University), Chongqing, China; ^2^The First Camp of Cadet Brigade, School of Basic Medicine, Third Military Medical University (Army Medical University), Chongqing, China

**Keywords:** oligodendrocytes, oligodendrocyte progenitor cells, NG2, mGFP, myelin dynamics

## Abstract

Chronic alcohol consumption causes cognitive impairments accompanying with white matter atrophy. Recent evidence has shown that myelin dynamics remain active and are important for brain functions in adulthood. For example, new myelin generation is required for learning and memory functions. However, it remains undetermined whether alcohol exposure can alter myelin dynamics in adulthood. In this study, we examine the effect of chronic alcohol exposure on myelin dynamics by using genetic approaches to label newly generated myelin (NG2-CreERt; mT/mG). Our results indicated that alcohol exposure (either 5% or 10% in drinking water) for 3 weeks remarkably reduced mGFP + /NG2- new myelin and mGFP + /CC1 + new oligodendrocytes in the prefrontal cortex and corpus callosum of 6-month-old NG2-CreERt; mT/mG mice as compared to controls without changing the mGFP + /NG2 + oligodendrocyte precursor cells (OPCs) density, suggesting that alcohol exposure may inhibit oligodendrocyte differentiation. In support with these findings, the alcohol exposure did not significantly alter apoptotic cell number or overall MBP expression in the brains. Further, the alcohol exposure decreased the histone deacetylase1 (HDAC1) expression in mGFP + /NG2 + OPCs, implying epigenetic mechanisms were involved in the arrested OPC differentiation. Together, our results indicate that chronic exposure to alcohol can inhibit myelinogenesis in the adult mouse brain and that may contribute to alcohol-related cognitive impairments.

## Introduction

Alcohol consumption has been linked to long-term and severe brain dysfunctions. For example, alcohol use disorders (AUDs) may lead to disrupted execution of motivated behaviors, declining memory capacity and increased stress (Zorumski et al., 2014; Vetreno et al., 2016). In addition to the alternations in neurotransmitter level and neuronal structural change (Davis and Wu, 2001; Romero et al., 2013; Abrahao et al., 2017), MRI imaging indicated alcohol-related abnormalities in the white matter (Monnig et al., 2015). Evidence of human and animal studies has revealed that white matter integrity is susceptible to alcohol exposure especially during fetal and adolescent stage (Pascual et al., 2014; Vargas et al., 2014; Rice and Gu, 2019). The white matter is composed of abundant axons wrapped with myelin sheaths, the multiple concentric cell membranes that are generated by oligodendrocytes (OLs) in the CNS. Myelin sheaths insulate axons and ensure fast action potential propagation along the axon (Zalc and Colman, 2000; Nave and Werner, 2014; Seidl, 2014).

Increasing evidence has shown myelin is undergoing dynamic changes in the adult CNS (Young et al., 2013; Hill et al., 2018; Hughes et al., 2018). The oligodendrocyte precursor cells (OPCs) are widely and evenly distributed in the adult mouse CNS, and these cells can divide or differentiate into mature OLs. The new myelin generation has been demonstrated important in regulating cognitive functions, such as learning and memory capacities (McKenzie et al., 2014; Pan et al., 2020; Wang et al., 2020; Xin and Chan, 2020). Given the importance of myelin dynamics in brain functions, it remains largely unknown how chronic alcohol exposure could affect myelin dynamics in adult mice.

In this study, we found chronic alcohol exposure significantly inhibited myelinogenesis by using cell-specific fluorescent labeling. The inhibited myelination is due to, at least partially, through an epigenetic mechanism. These findings suggest that disrupted myelin dynamics may contribute to alcohol-related cognitive impairments.

## Materials and Methods

### Mice

The NG2-CreERt (The Jackson Laboratory, Catalog # 008538) mouse strain has been described in a previous study (Wang et al., 2020). CreERt expression is under the transcriptional control of the regulatory sequences of the *cspg4* gene. The mT/mG mice (The Jackson Laboratory, Catalog # 007676) were on a C57BL/6J congenic background. Prior to Cre recombination, cell membrane-localized expression of the tdTomato (mT) fluorescence reporter is observed in a wide range of cells/tissues. Cre recombinase-expressing cells (and future cell lines derived from these cells) express the cell membrane-bound EGFP (mG) fluorescence reporter, replacing the red fluorescence. The mT/mG mice were crossed with the NG2-CreERt mice line to generate NG2-CreERt; mT/mG mice, which enabled us to observe the target cells and the recombination efficiency of NG2-CreERt. Genotypes of all mice were determined using a PCR analysis of tail genomic DNA with appropriate primers. Male and female mice were used for all experiments without bias. All the mice were housed in temperature- and humidity-controlled environment with free access to standard chow and water on a 12 h/12 h light/dark cycle.

### Ethics Statement

All animal experiments were performed according to an approved protocol from the Laboratory Animal Welfare and Ethics Committee of the Third Military Medical University.

### Tamoxifen Administration

Tamoxifen (Sigma-Aldrich, St. Louis, MO, United States) was dissolved in sunflower oil to a concentration of 10 mg/ml by shaking the solution for 3 h at 37°C. For CreERt-mediated recombination, mice received 5 μl per gram body weight by oral gavage for seven consecutive days.

### Alcohol Treatment Model

**FIGURE 1:**
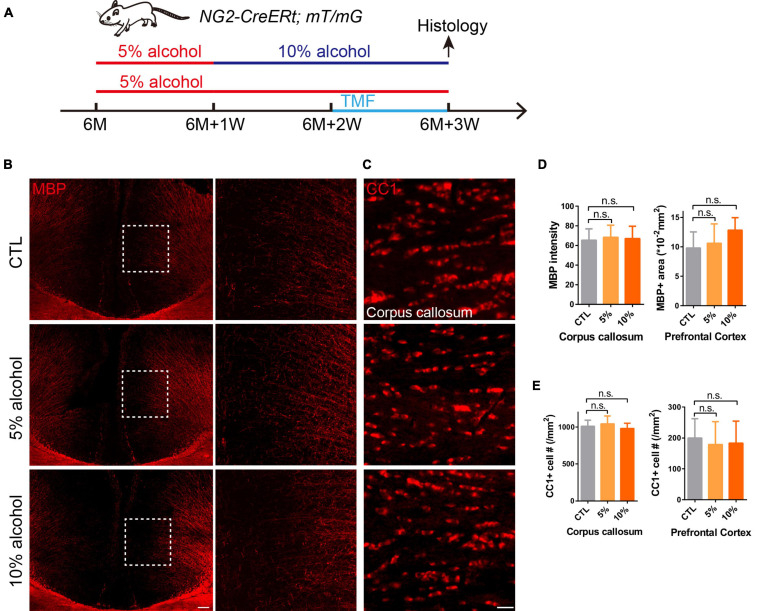
Overall myelin and mature OLs are not affected after chronic alcohol exposure. **(A)** Schematic diagram displaying the time course for alcohol (5 or 10%) treatment, tamoxifen induction and histology in the NG2-CreERt; mT/mG mice. Note that in the 10% alcohol group, 5% alcohol was administrated in the first week as an adaption step. **(B)** MBP staining showing intact myelin sheath in the cortex of alcohol treated groups compared to littermate controls. Scale bar 200 μm. The dotted white box was magnified in the right panel. **(C)** Immunostaining of CC1 showing comparable number of mature OLs in the corpus callosum of mice in alcohol treated and CTL group. Scale bar 20 μm. **(D)** Quantification of MBP + area and MBP intensity in the prefrontal cortex and corpus callosum, respectively (n.s. *p* = 0.32 and 0.95, *n* = 4). **(E)** Quantification of CC1 + cell number in the corpus callosum and prefrontal cortex (n.s. *p* = 0.85 and 0.47, *n* = 6).

Six-month old mice were divided into three groups: control group, 5% alcohol (v/v) (Chuandong huagong, Chongqing, ≥99.7%) group and 10% (v/v) alcohol group. As shown in Figure 1A, in the first week mice in experimental groups were all treated with 5% alcohol, while in the remaining 2 weeks, 5% alcohol (v/v) and 10% (v/v) alcohol were used as the only source of water in the 5% group and 10% group, respectively. Mice in the CTL group were administrated with normal drinking water during the whole process.

### Tissue Processing

Mice were deeply anesthetized with 1% pentobarbital and transcardially perfused with 4% paraformaldehyde (PFA) in PBS. Brains were dissected and dehydrated in 30% sucrose at 4°C. Serial coronal sections (20 μm) were obtained using a cryostat microtome (MS 1900, Leica, Wetzlar, Germany).

### Immunofluorescent Staining

Free floating sections were blocked with 1% bovine serum albumin (BSA) and 0.5% Triton-X 100 for 1 h at room temperature and then sequentially incubated with primary antibodies overnight at 4°C and the fluorescent-dye-conjugated secondary antibodies for 2 h at room temperature. Primary antibodies included: rabbit anti-NG2 (1:200, Millipore, Temecula, CA, United States), goat anti-MBP (1:500, Santa Cruz Biotechnology, Dallas, TX, United States), mouse anti-CC1 (1:500, EMD Millipore, Darmstadt, Germany), rabbit anti-HDAC1 (1:200, Invitrogen, Cat: # PA1-860), goat anti-GFAP (1:500, Santa Cruz, Cat: sc-6170), rabbit anti-Iba1 (1:600, Wako, Cat: 019-19741), mouse anti-Vimentin (1:500, Thermo, Cat. # MS-129-R7), Rat anti-CD68 (1:500, abcam, ab53444). Appropriate Alexa Fluor-conjugated secondary antibodies included: donkey anti-mouse, rabbit anti-goat, donkey anti-rat, donkey anti-rabbit IgG (1:1,000, Life Technologies, Carlsbad, CA, United States). DAPI (1:1,000, Invitrogen).

### Tunel Staining

To detect apoptosis, the TUNEL (terminal deoxynucleotidyl transferase dUTP nick end labeling) assay was performed following the manufacturer’s instructions (Invitrogen, C10619). Briefly, floating sections were washed twice with PBS and permeabilized with proteinase K solution. The sections were incubated with the TdT (termial deoxynucleotidyl transferase) reaction buffer, containing EdUTP and the TdT enzyme, at 37°C for 1 h in a humidified atmosphere. The sections were then washed and incubated in the fresh Click-iT^TM^ Plus Tunel reaction cocktail at 37°C for 30 min in a dark humidified box. At last, brain sections were washed with PBS to remove unincorporated fluorescent substrate. The specimens were observed with a fluorescence microscope with an excitation wavelength of 647 nm to detect Tunel positive cells.

### Image Acquisition and Quantification

Fluorescent images were captured using a spinning disk confocal super resolution microscope (Olympus, SpinSR10, Shinjuku, Tokyo) or a VS200 Research Slide Scanner (Olympus), with excitation wavelengths appropriate for Alexa Fluor 488 (488 nm), 596 (568 nm), 647 (628 nm), or DAPI (380 nm). Morphometric analysis was performed using the Sholl analysis by Image-J by using standard concentric circles to evaluate the diameter and area of microglia and astrocytes (Niu et al., 2012). Briefly, the program superimposes a grid of some concentric circles from the soma with increasing radii, and then measures the number of intersections and the membrane expansion generated by the glials with each circle. For the statistical analysis, at least three representative fields (20 ×) were acquired from each sample. Detection and quantification were performed using Image-J 5.0 software (Media Cybernetics, Silver Spring, MD, United States^1^).

### Statistic Analysis

Data in all figures were presented as means ± S.E.M. The statistical significance of differences between groups was determined using one-way ANOVA followed by *post hoc* Tukey test for multiple comparisons. Significance was reported as ^∗^*p* < 0.05, ^∗∗^*p* < 0.01 or ^∗∗∗^*p* < 0.001.

## Results

### Chronic Alcohol Exposure Dose Not Cause Overt Myelin Loss in Adult Mouse Brains

Since the adult CNS is enriched with dense myelin sheaths, we set out to examine whether chronic alcohol exposure could change myelin density, the 6-month-old mice were introduced to alcohol for 3 weeks (Figure 1A). We immunostained for MBP on the brain sections with or without alcohol exposure and our result indicated that the MBP + myelin area or intensity was not significantly altered in the cortex and corpus callosum of the 5 or 10% alcohol treated brains as compared to the vehicle controls (Figures 1B,D), suggesting that chronic alcohol exposure may not cause overt myelin loss. In support with this finding, the number of CC1 positive OLs was not significantly altered in the brain sections with alcohol exposure, in relative to the controls (Figures 1C,E). These results indicate that 5 or 10% alcohol exposure for 3 weeks does not cause significant loss of myelin or OLs.

### Myelinogenesis Is Inhibited by Chronic Alcohol Exposure in Adult Mouse Brains

**FIGURE 2:**
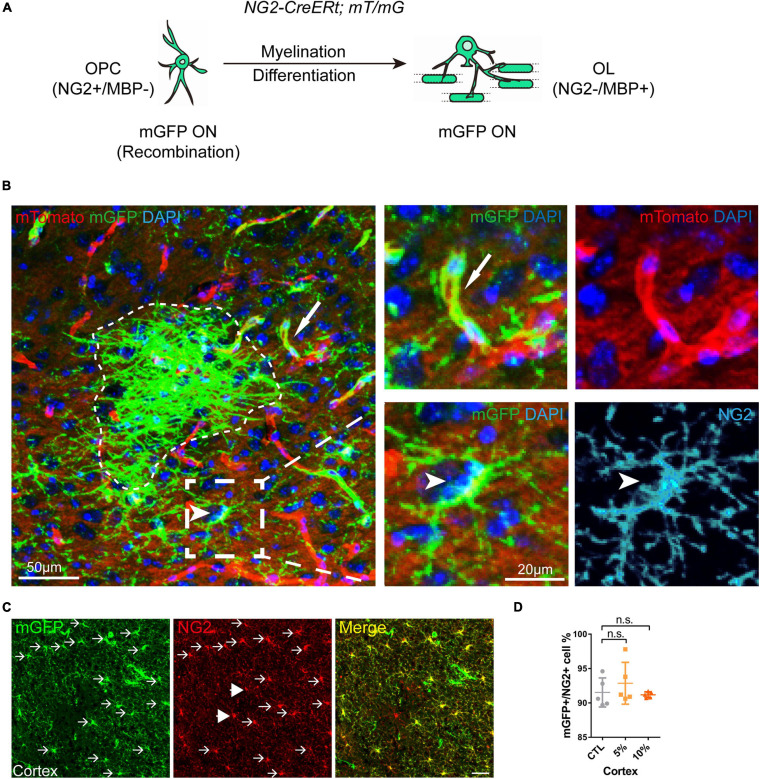
Visual representation of the mGFP positive cells in NG2-CreERt; mT/mG mice. **(A)** Schematic illustration showing the mGFP expression pattern in the NG2-CreERt; mT/mG mice. **(B)** Representative images showing mGFP (green)/NG2 (cyan) double positive OPCs (arrowhead), mGFP + NG2- new OLs or myelin sheaths (dotted irregular line) and mGFP + pericytes (arrows) attached to mTomato positive (red) blood vessels. **(C)** Immunostaining of NG2 showing the NG2/mGFP double positive cells (arrows) and NG2 + mGFP- cells (arrowheads). Scale bar 50 μm. **(D)** Quantification of the percentage of mGFP (green) positive cells in the NG2 (red) positive cells (OPCs) (n.s. *p* = 0.45, *n* = 5).

**FIGURE 3:**
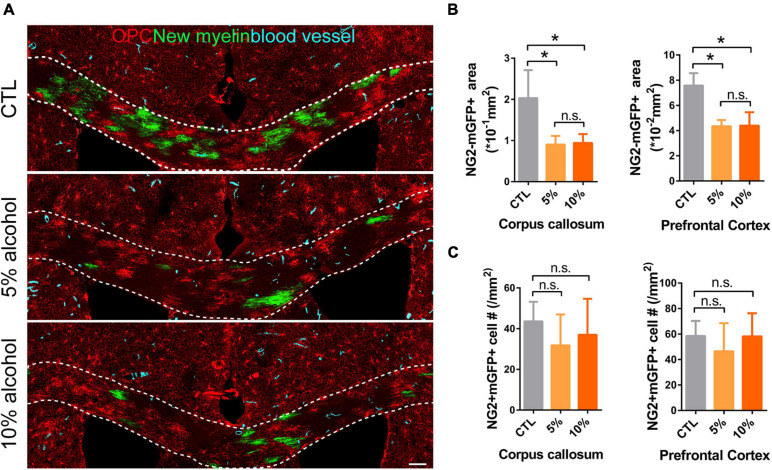
Myelin generation is inhibited by chronic alcohol exposure in adult mice. **(A)** Representative images of OPCs (red), newly formed OLs (green) and blood vessels (cyan) showing remarkably decreased myelin generation in alcohol treated groups. Scale bar 100 μm. **(B)** Quantification of NG2-mGFP + myelin area in the corpus callosum and prefrontal cortex. [*p < 0.05, n.s. Tukey (5 vs. 10%) *p* = 0.99 and 0.99, *n* = 6] **(C)** Quantification of NG2 + mGFP + cells in the corpus callosum and prefrontal cortex (n.s. *p* = 0.46 and 0.49, *n* = 5).

**FIGURE 4:**
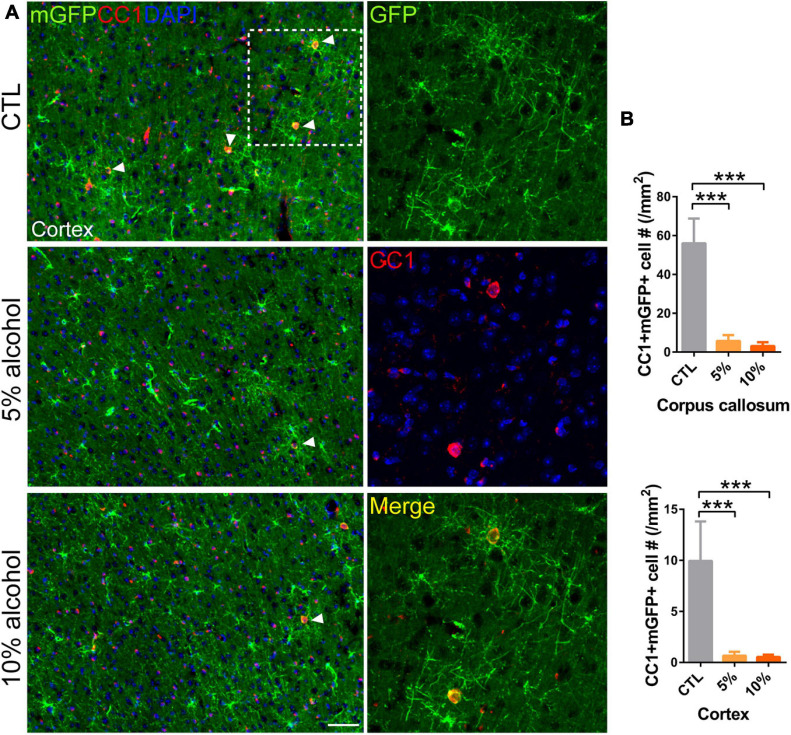
Mature OLs generation is reduced by alcohol drinking in adult mice. **(A)** Immunostaining of CC1 in the cortex showing the newly generated mature OLs (CC1 + mGFP +) (arrowheads). Scale bar 50 μm. The dotted white box was magnified in the right panel. **(B)** Quantification of CC1 + mGFP + new OLs in the corpus callosum and prefrontal cortex (***p < 0.001, *n* = 7).

Increasing evidence has shown that myelinogenesis remains active in the young and middle-aged mouse brains, which is important for cognitive functions (Pan et al., 2020; Steadman et al., 2020; Xin and Chan, 2020). To understand if alcohol could alter myelin generation, we utilized the transgenetic fluorescent reporter mouse line (NG2-CreERt; mT/mG), in which OPCs started to express membrane bound green fluorescent protein (mGFP) upon tamoxifen induction. Upon differentiation, mature OLs and associated myelin sheaths are also visible and express mGFP (Figure 2A). Blood vessels highly express mT (tomato) and can be visualized in the red channel. By immunostaining for NG2, we can divide the mGFP positive cells into three types: OLs (NG2-/mGFP +), OPCs (NG2 + /mGFP + /mT-) and pericytes (NG2 + /mGFP + /attached to blood vessels) (Figure 2B). We first confirmed that the recombination was specific and the efficiency was around 91% in three groups as shown (Figures 2C,D). Then we pseudo-colored blood vessels in cyan and OPCs in red to clearly visualize newly formed myelin (green) as we previously did (Figure 3A; Wang et al., 2020). We quantified the area of mGFP + new myelin in prefrontal cortex, corpus callosum and hippocampus. Our results indicated a remarkable decrease of mGFP + new myelin in the alcohol (5 and/or 10%) groups in those brain regions as compared to the vehicle controls (Figures 3A,B). In line with this change, we found that the number of newly generated mature OLs (CC1 + /mGFP + cells) was also obviously decreased in the alcohol exposure groups as compared to the vehicle controls (Figures 4A,B). It is noted that the effects on myelinogenesis seems to be similar between 5% and 10% alcohol groups (Figures 3A,B), suggesting the inhibitory effect on new myelin formation is not dependent on the concentration of consumed alcohol.

### Chronic Alcohol Exposure Does Not Change OPC Density or Induce Cell Apoptosis

**FIGURE 5:**
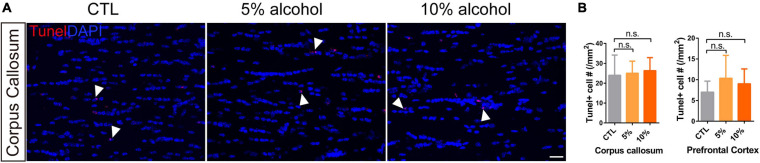
Chronic alcohol treatment does not change cell apoptosis level in adult mice brain. **(A)** Tunel staining showing a few apoptotic cells (arrowheads) in the corpus callosum. Scale bar 20 μm. **(B)** Quantification of Tunel positive cell in the corpus callosum and prefrontal cortex (n.s. *p* = 0.93 and 0.62, *n* = 3).

To understand if the inhibited myelinogensis is related to OPC density, we counted OPC (NG2 + /mGFP +) number in the brains. Our results indicated that OPCs density was not significantly altered in corpus callosum and prefrontal cortex by alcohol exposure (Figures 3A,C), suggesting the inhibited new myelin formation is not due to the insufficiency of OPC population. To examine if alcohol could increase cell apoptosis, we utilized the TUNEL assay to label apoptotic cells and only found very few TUNEL + cells in the brain sections from either alcohol or vehicle treated group. No significant difference was detected between alcohol treated brain (5 and 10%) and the vehicle controls (Figures 5A,B). These findings demonstrate that the decreased myelinogenesis caused by alcohol is not due to the OPC density nor the apoptotic cells.

### Alcohol Exposure Does Not Activate Astroglia or Microglia

**FIGURE 6:**
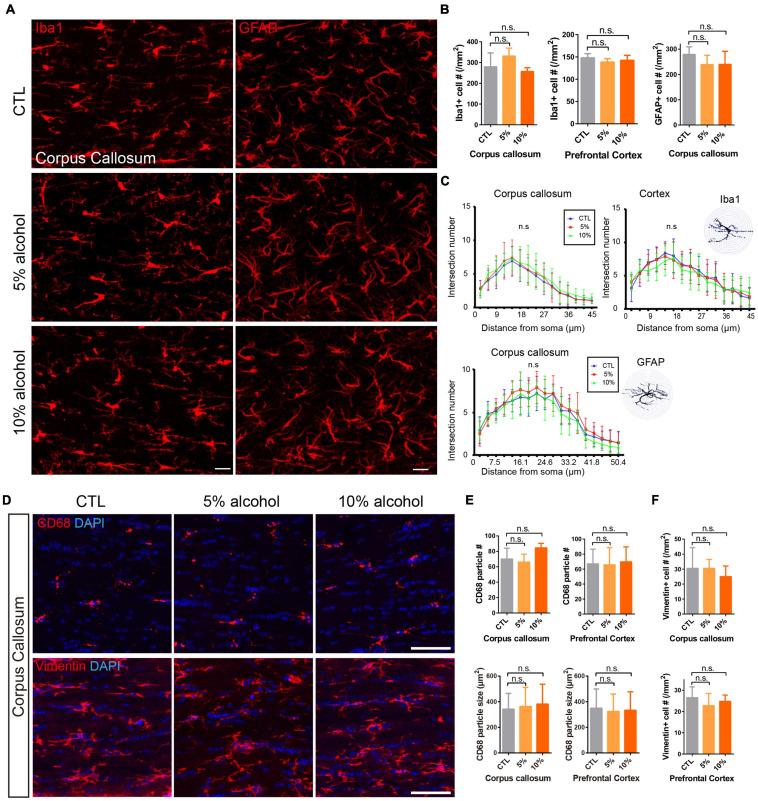
Alcohol exposure does not activate astroglia or microglia. **(A)** Immunostaining of GFAP and Iba1 showing astrocytes and microglial cells in the corpus callosum. Scale bar 20 μm. **(B)** Quantification of Iba1 positive cells in the corpus callosum and prefrontal cortex (n.s. *p* = 0.43 and 0.33, *n* = 4) and GFAP positive cells in the corpus callosum. (n.s. *p* = 0.11, *n* = 3) **(C)** The Sholl analysis showed comparable morphology of microglia and astrocytes in the corpus callosum and cortex in CTL and alcohol treated groups. **(D)** Immunostaining of CD68 and Vimentin showing astrocytes and microglial cells in the corpus callosum. Scale bar 50 μm. **(E)** Quantification of the number of CD68 positive cells and the size of CD68 particles in the corpus callosum and prefrontal cortex. [n.s. p (number) = 0.08 and 0.96, p (size) = 0.07 and 0.12, n = 4] **(F)** Quantification of the number of Vimentin positive cells (n.s. *p* = 0.65 and 0.55, *n* = 4).

Inflammatory microenvironments could inhibit myelinogenesis (Li et al., 2020), which has been reported in alcohol consumption animal models (Pascual et al., 2014; Erickson et al., 2019). We next assessed the density and morpohology of astroglia and microglia after alcohol exposure. We did immunostaining for GFAP or Iba1 on the brain sections. Our results indicated that neither the density nor the morphology of the GFAP or Iba1 positive cells was altered after alcohol exposure (Figures 6A–C). More specifically, Vimentin and CD68 staining was performed to label activated astroglia and microglia, respectively. It also revealed that the density of activated astroglia and microglia was not significantly altered by alcohol treatment (Figures 6D–F). In addition, the size of CD68 positive particles in the microglial plasma was comparable between CTL and alcohol treated groups (Figures 6D,E). This further confirmed that astrogliosis and microglial phagothytosis were not active in the brain of alcohol treated mice (Safaiyan et al., 2016; Wilhelmsson et al., 2019). Those results suggest that the inhibited myelinogenesis is unlike a result from the overactivation of microglial cells or astrocytes.

### Reduced HDAC1 Expression in OPC by Alcohol Exposure

**FIGURE 7:**
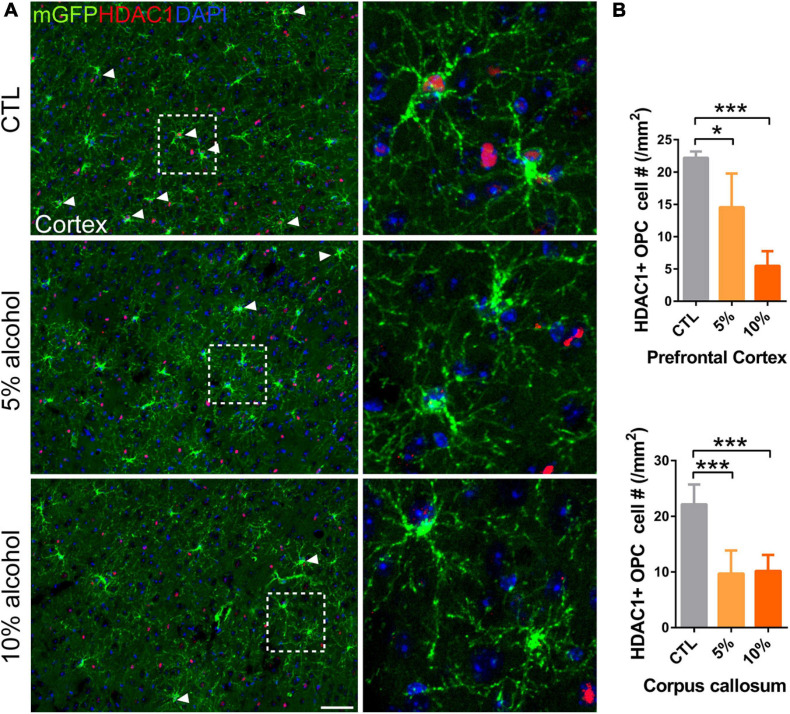
HDAC1 expression is reduced in adult OPCs by alcohol exposure. **(A)** Immunostaining of HDAC1 showing HDAC1 + GFP + OPCs (arrowheads) was remarkably reduced in the cortex of alcohol-treated mice. The dotted white box was magnified in the right panel. Scale bar 50 μm. **(B)** Quantification of HDAC1 + GFP + OPCs in the corpus callosum and prefrontal cortex (*p < 0.05, ***p < 0.001, *n* = 6).

Oligodendrocyte precursor cell differentiation is a complex process that is orchestrated by a number of mechanisms (Emery, 2010; He and Lu, 2013). Upon the initiation of differentiation, OPCs need to change morphologically and transcriptionally. Thus, the epigenetic regulators are recognized as an important indicator for OPC differentiation (Huang et al., 2015; Lu et al., 2019). Histone deacetylases (HDACs) are responsible for histone acetylation, and the expression of HDACs is linked to transcriptional repression of a number of myelination inhibitory genes (Shen et al., 2008). Given that the inhibited myelinogenesis might be associated with the HDAC1 level, We speculated that the HDAC1 expression could not be up-regulated and that hinders the initial of differentiation. As expected, immunofluorescent staining for HDAC1 showed that the expression of HDAC1 in mGFP + OPCs was remarkably reduced in alcohol treated group (Figures 7A,B), implying suppressed expression of myelin-related genes in OPCs.

## Discussion

Chronic alcohol exposure causes brain dysfunctions, i.g., cognitive deficits, emotional and behavioral changes (Zorumski et al., 2014; Vetreno et al., 2016). It has been shown that chronic alcohol consumption is associated with loss of white matter in the brain at all stages of life (Alfonso-Loeches et al., 2012; Vargas et al., 2014), but the role alcohol administration on myelin dynamics remains unknown. Recent evidence has linked myelin dynamics with cognition, motor learning and spatial memory (Xiao et al., 2016; Steadman et al., 2020; Xin and Chan, 2020). In the present study, we applied 5 and 10% alcohol treatment for 3 weeks, representing a mild and moderate levels of alcohol intoxication, respectively (He et al., 2007; Alfonso-Loeches et al., 2012; Pascual et al., 2014; Vargas et al., 2014; Tavares et al., 2019). Our results demonstrated that a mild level of alcohol exposure is enough to inhibit OPC differentiation and new myelin formation in the adult mice.

Though myelin is very dense in the white and gray matters of the adult brains, new myelin sheaths are continuously generated *via* OPC differentiation. The newly added OLs can replace apoptotic OLs on one hand. On the other hand, there are still a large amount of axons remaining unmyelinated in most brain areas such as corpus callosum and prefrontal cortex (Young et al., 2013). Deposition of new myelin on the denuded axons may modulate the functions of neuronal circuits, as a form of neuroplasticity (Kaller et al., 2017). Recent studies have shown the active myelinogenesis in adult mouse brains is necessary for motor learning, memory consolidation and preservation, and cognitive functions (Xiao et al., 2016; Pan et al., 2020; Steadman et al., 2020). Diminished myelin formation in aged brains contributes to declining memory capacities (Wang et al., 2020). We demonstrated that 5% alcohol exposure for 3 weeks is enough to cause remarkable inhibitory effects on myelinogenesis, suggesting that myelinogenesis is sensitive to alcohol in adulthood. This findings is in support with previous studies showing exposure to 10% alcohol for only 2 weeks at prenatal stage or during adolescence induces myelin developmental abnormalities (Vargas et al., 2014; Newville et al., 2017).

Oligodendrocyte differentiation and myelination is orchestrated by a net of regulators (Emery, 2010). Our result showed that the expression of HDAC1 in new adult OPCs was obviously decreased, indicating disrupted epigenetic modulation after alcohol treatment may hinder the OPC differentiation program. In line with our findings, previous studies have shown that the recruitment of HDACs is necessary for remyelination in aged mice (Shen et al., 2008; Ye et al., 2009). It is noticeable we did not detect significant changes of MBP positive myelin and CC1 positive cells in this case, although myelinogenesis is inhibited by alcohol. One possibility is that we examined new myelin that was generated for only 1 week and the newly formed OLs and associated myelin segments only occupy less than 5% percentage of total OLs and myelin. In this premise, our results did not detect overt myelin loss, suggesting that the pre-existed myelin may be much less sensitive to alcohol for 3 weeks and probably, a much longer time of alcohol exposure may damage pre-existing myelin and cause degeneration. In line with this notion, studies have found obvious changes in myelin structure after 5 month to 1 year of 10% alcohol exposure in adult mice (He et al., 2007; Alfonso-Loeches et al., 2012). Previous studies have shown that adolescent binge drinking could lead to myelin damage in the PFC and disrupted working memory (Vargas et al., 2014). Even maternal alcohol binge drinking will induce persistent neuroinflammation associated with myelin protein loss and offspring mice present motor coordination impairments (Cantacorps et al., 2017). Here in our model, despite that the overall myelin structure and mature OLs is not affected by alcohol consumption, it is plausible that the cognitive and motor functions are disturbed, as myelinogenesis is proven to be necessary for motor leaning/coordination and memory capacity (Xiao et al., 2016; Pan et al., 2020; Steadman et al., 2020; Wang et al., 2020; Chen L. et al., 2021).

It remains unclear how alcohol can inhibit myelinogenesis. Alcohol can pass through blood brain barrier and be concentrated in the brain. Experiments have shown that alcohol can directly bind a range of molecular targets in the brain (Abrahao et al., 2017). Besides, acetaldehyde and acetate, two metabolites of alcohol, can be transported into the brain as well (Coutts and Harrison, 2015; Jamal et al., 2016). An *in vitro* study has shown that OPC differentiation and myelin formation is much more sensitive to acetaldehyde than alcohol. In addition, a recent study reported that acetate could change epigenetics in the brain (Mews et al., 2019), but it is still unknown whether these metabolites could affect oligodendroglial differentiation *in vivo*. Meanwhile, alcohol display universal effects in the CNS, such as altering neuronal function and astroglial gene expression (Miguel-Hidalgo, 2018; Jin et al., 2021). These effects could also indirectly affect the differentiation of adult OPCs. Despite the unknown mechanisms, it is feasible to rescue inhibited myelinogenesis in adult brains, as a number of FDA- approved drugs have been shown efficient in enhancing myelin generation in adult mice (Mei et al., 2016; Chen J. F. et al., 2021). Promoting OPC differentiation and myelination may be beneficial for the treatment of alcohol related cognitive impairments.

## Data Availability Statement

The original contributions presented in the study are included in the article/supplementary material, further inquiries can be directed to the corresponding authors.

## Ethics Statement

The animal study was reviewed and approved by Laboratory Animal Welfare and Ethics Committee of the Third Military Medical University.

## Author Contributions

FG, Y-FZ, TL, XH, KL, R-XL, and S-YW performed the experiments. FG, Y-FZ, XH, KL, and TL analyzed the data. FW and LX contributed to the revision of the manuscript and gave pertinent opinions. TL and FM provided intellectual contributions and wrote the manuscript. All authors read and approved the manuscript.

## Conflict of Interest

The authors declare that the research was conducted in the absence of any commercial or financial relationships that could be construed as a potential conflict of interest.

## Publisher’s Note

All claims expressed in this article are solely those of the authors and do not necessarily represent those of their affiliated organizations, or those of the publisher, the editors and the reviewers. Any product that may be evaluated in this article, or claim that may be made by its manufacturer, is not guaranteed or endorsed by the publisher.

## Footnotes

1
https://imagej.nih.gov/ij/
